# Place Attachment and Household Disaster Preparedness: Examining the Mediation Role of Self-Efficacy

**DOI:** 10.3390/ijerph18115565

**Published:** 2021-05-23

**Authors:** Ziyi Wang, Ziqiang Han, Lin Liu, Shaobin Yu

**Affiliations:** 1 School of Political Science and Public Administration, Shandong University, Qingdao 266237, China; ziyi.wang.em@mail.sdu.edu.cn (Z.W.); ziqiang.han@sdu.edu.cn (Z.H.); liulinsdu@sdu.edu.cn (L.L.); 2 Institute of Governance, Shandong University, Qingdao 266237, China

**Keywords:** place attachment, self-efficacy, disaster preparedness, disaster experience, China

## Abstract

Household preparedness is essential for resilience-building and disaster risk reduction. Limited studies have explored the correlations between place attachment, self-efficacy, and disaster preparedness, especially in the east Asian cultural context. This study investigates the mediating role of self-efficacy between place attachment and disaster preparedness based on data from the 2018 Shandong General Social Survey (N = 2181) in China. We categorized the preparedness behaviors into three specific clusters: material, behavioral and awareness preparedness. Multiple linear regressions and the Sobel Goodman tests were employed to estimate the correlations with the control of necessary confounding variables such as disaster experience, socioeconomic and demographic characteristics. The results demonstrate that both the place attachment and self-efficacy are correlated with higher degrees of overall preparedness and all three types of preparedness, and self-efficacy plays a mediating role between place attachment and disaster preparedness. These findings highlight the importance of promoting place attachment and self-efficacy in the advocacies and outreach activities of disaster preparedness.

## 1. Introduction

Disaster preparedness, as the knowledge and capacities developed by institutions, communities, and individuals to anticipate, respond to, and recover from the impacts of all disasters and the related efforts of increasing such knowledge and capacities [1], is essential to reduce the impact of a disaster. Pre-disaster risk reduction efforts include both mitigation and preparedness activities. Some scholars and practitioners differentiate the two concepts. They suggest that mitigation activities are related to the physical and engineering efforts and long-run solutions (e.g., building sea walls) [2,3], while the preparedness activities are more about the knowledge and capacity building activities, but some other researchers treat all the pre-event mitigation and preparedness activities as similar concepts [4,5]. Disaster preparedness behaviors include all the actions taken to reduce the potential impact of potential disasters. In general, disaster activities can be divided into material preparedness (e.g., preparing an emergency kit at home), awareness or knowledge preparedness (e.g., learning knowledge about disasters), and behavioral preparedness (e.g., participating in exercise or drills, being a volunteer) [6,7], and during the emergent situation, information seeking, emotional coping, and the adoption of protective actions (e.g., emergency evacuation) are the general clusters of preparedness behaviors [8]. Regarding the entities of disaster preparedness, they can be implemented either by individuals/households or organizations such as government agencies [9,10,11] or business companies [12]. Previous calculation using data from the United States of America indicated that one dollar of investment in pre-disaster mitigation and preparedness efforts could prevent six dollars in losses from potential disasters [13]. Since the “whole community” approach is suggested and all stakeholders are encouraged to engage in disaster preparedness [14], the disaster preparedness of individuals and households, which are the basic social unit and the very frontline of disaster response, deserve to be further investigated.

Scholars have developed or adopted various theoretical frameworks to understand the predictors and barriers of preparedness behaviors in the face of risk, such as the protective action decision model, health belief model, extended parallel process model, theory of planned behavior, social cognitive theories, and personal-relative-to-event model, etc., and all these frameworks were concentrated in the social-psychological and behavioral science domain [6,15]. The social-cognitive framework highlights the importance of place attachment, types of efficacy, and perceived responsibility among stakeholders in predicting the adoption of preparedness behaviors, but the effects of these variables in individual and household disaster preparedness are insufficiently investigated in empirical studies [15,16]. Therefore, inspired by the social cognitive framework in disaster studies [16], we developed this study by investigating the complex relationships between place attachment, self-efficacy, and disaster preparedness behaviors.

Place attachment refers to the affect and emotions that connect people to places or physical environment [17,18]. It can influence an individual’s intention to prepare or the actual preparedness behaviors, especially in times of stress. Place attachment is a crucial concept widely used in environmental studies and adopted in cross-disciplinary natural hazards research. However, the effects of place attachment on risk perception and disaster preparedness varied in different cultural and hazard contexts in current studies. Bonaiuto’s review of 31 studies investigating the correlations between place attachment and natural hazards risk perceptions found that there were both positive and negative relations between place attachment and risk perception, place attachment, and risk coping behaviors [19]. Moreover, place attachment can affect the risk perception and coping behaviors in multiple ways, either directly or indirectly, as moderating or mediating roles [18,19,20].

Place attachment can drive individuals’ personal emotions into practical actions that protect themselves and their communities [20]. This assumption was supported in India regarding flood preparedness [18] and in southwest China regarding insurance purchasing intention toward landslides [21]. Nevertheless, a more substantial place attachment may lead to underestimating potential risks [22,23], or unwillingness to relocate, or a greater likelihood of returning to risky areas after a natural disaster [19,24]. The effect of place attachment on individuals’ risk perceptions and risk coping behaviors can be mediated by variables such as longevity in or the familiarity with a place [25]. It can also be moderated by variables such as the environmental contexts or the types of attachments. For example, in a study about wildfire mitigation and preparedness in Australia, place attachment can only motivate the residents’ preparedness actions in the rural sample, but not in the urban and the wildland–urban interface samples [26]. A similar study about flood preparedness in Orissa, India, also revealed that although genealogical and economical attachment to a place contributed to flood preparedness, religious attachment did not [18].

Self-efficacy is considered as an individual’s belief or perception about his/her capacity to practice or implement a task or action [27]. Generally, collective efficacy, response/outcome efficacy are similar efficacy concepts used in literature along with self-efficacy. The response efficacy [28], also termed as outcome efficacy [29,30], refers to the belief or perception of the usefulness or effectiveness of the protective activities or the adaptative behaviors. Similarly, collective efficacy refers to the belief or perception of a group’s conjoint capabilities to organize or do something [31,32]. In the field of disaster research, self-efficacy refers to the assessment of one’s own ability to initiate or complete a preventive, protective, or adaptive behavior [29]. Self-efficacy is an essential social cognitive precursor to prepare for disasters in the social-cognitive theory model [16]. People will develop intentions to prepare for disasters only if they have adequate expectations about being able to perform the act [33]. Most studies have demonstrated that high self-efficacy can motivate disaster preparedness intentions or the actual behaviors [30,34,35], or the specific protective actions in emergencies such as emergency evacuation [29]. Such positive effects were primarily observed in preparation for floods [36,37,38,39], earthquakes [40], or climate change-related hazards [30]. In household disaster preparedness studies, self-efficacy is always captured by the self-reported confidence of their capacity for implementing a protective action against a disaster or successfully coping with potential disasters [33,39]. It appears that the role of self-efficacy in disaster preparedness is still relatively understudied in terms of geographical, social and cultural diversity, though there is an increasing trend in recent years [30].

The correlation between place attachment and self-efficacy has also been examined a limited amount in the context of disaster risk perception and preparedness studies, because most of the studies have not yet linked the two together. According to the place identity theory, place attachment can produce a stronger sense of self-efficacy [41] because the environment maintains the feeling of self-efficacy facilitation [42]. The familiarity and attachment to a place may make people feel unique, in control of, and good about themselves [43], which eventually provides feelings of distinctiveness, continuity, self-esteem, and self-efficacy [44].

**Figure 1:**
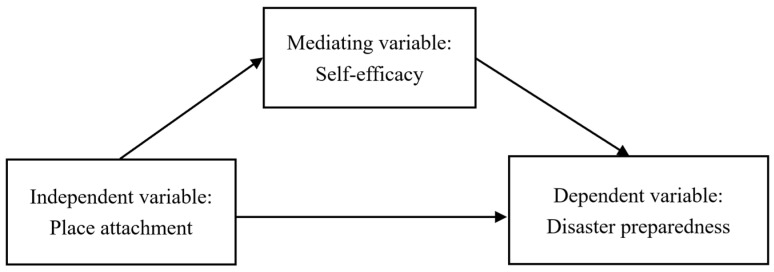
The proposed conceptual model.

**Figure 2:**
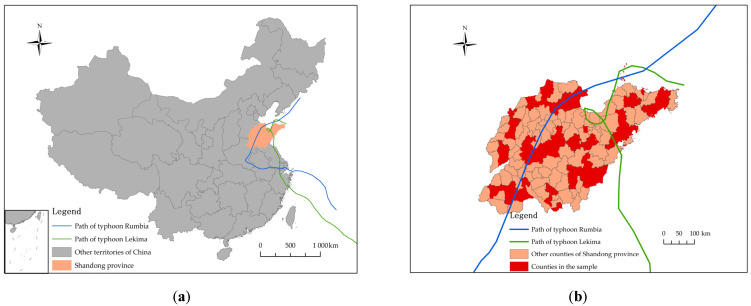
(**a**) The location of Shandong province and paths of Typhoon Rumbia and Lekima; (**b**) Sampled counties in Shandong province and paths of Typhoon Rumbia and Lekima. Note: Data source of typhoon path is from China Meteorological Administration tropical cyclone database. This figure was prepared with ArcGIS 10.7 (ESRI, Redlands, CA, USA).

Therefore, guided by the social-cognitive theory, this study aims to investigate the correlations between place attachment, self-efficacy, and household preparedness using a representative survey conducted in 2018 in Shandong province, China. This study can enrich the current knowledge by (1) linking the place attachment, self-efficacy, and disaster preparedness in one model, and exploring their complex relations, as shown in Figure 1; (2) testing these relationships in the context of a place with fewer disasters before but facing increasing threats from climate-related disasters. Furthermore, the findings of this research can improve the social cognitive theory in disaster preparedness studies and eventually promote the individual and household’s disaster preparedness activities by promoting their confidence in protecting themselves (efficacy) from potential disasters. Based on the discussions above, we assume that self-efficacy can play a mediating role between place attachment and preparedness; thus, we hypothesize that:

**Hypothesis** **1** **(H1).** *Place attachment is positively correlated with household preparedness.*

**Hypothesis** **2** **(H2).** *A higher degree of self-efficacy predicts a higher degree of preparedness.*

**Hypothesis** **3** **(H3).** *Self-efficacy mediates the relationship between place attachment and disaster preparedness.*

## 2. Methods

### 2.1. Study Area and Participants

Data used in this analysis comes from a representative survey from Shandong province. As a coastal province of China, Shandong severely suffered from flood risk about 100 years ago due to the unstable situation of the Huang River [45]. However, during the decades after the establishment of the People’s Republic of China in 1949, the province has experienced much fewer occurrences of natural-induced disasters [46]. Nevertheless, more and more typhoons have hit this area in recent years. In 2018, the typhoon Rumbia hit Shandong province, followed by another typhoon, Lekima, in 2019 [47,48,49], as shown in Figure 2a,b. Typhoon Rumbia affected more than 1.47 million residents and caused a direct economic loss of about 9.2 billion Chinese Yuan (about 1.3 billion US dollars) [50]. Likewise, typhoon Lekima affected more than 1.66 million residents; among them, 183,800 had to be evacuated. It was estimated that direct economic loss was about 1.5 billion Chinese Yuan (about USD $212 million) due to the collapse of houses and the losses of agricultural productions [51]. In this scenario, studies about residents’ preparedness behaviors based on data from Shandong province are precious because the public has not experienced disasters for quite a long time, but the prospect of disasters looms large, especially typhoons and related floods.

The Shandong General Social Survey (SGSS) is a large-scale household survey project conducted by Shandong University, and the disaster preparedness module was included in the 2018 survey. The survey used a PPS (probability proportionate to size sampling) sampling strategy, a stratified, four-stage nonprobability sampling method. The primary sampling unit was the county, the second was the town, and the third was communities. Households were then randomly selected within the community using the household registration list. Residents aged 18 and above were the targeted population. We recruited 7382 households, and 4259 individuals in 4259 households responded to our survey, indicating a response rate of 57.69%. Since the disaster preparedness module was only included in one of the two versions of the questionnaire, this data included 2181 participants from 2181 households. Data collection was conducted through face-to-face interviews by trained college students between 26 May 2018 and 9 October 2018, with the assistance of the computer-assisted personal interviewing (CAPI) system. Finally, 1863 valid observations were included in our analysis after the dropping of records with missing values.

### 2.2. Measures

#### 2.2.1. Disaster Preparedness

Based on prior studies [6,7,52], 18 questions about disaster preparedness activities were incorporated into the survey. Specifically, seven of the questions were related to material preparedness (food, water, flashlight, emergency kit, radio, medicine, special needs) within a household, another seven about their planning and actions linked to disaster risk reduction (behavioral preparedness), and the last four about the participant’s awareness of disaster protective actions (awareness preparedness). The seven types of behavioral preparedness activities were “developing a written family emergency plan”, “having a reunion plan within family members for potential emergencies”, “paying attention to disasters related information”, “purchasing accident insurance for family members”, “participating in emergency training”, “discussing with friends and family members about what to do if emergencies happened”, and “being a volunteer or a member of community emergency response team”. The four awareness preparedness activities were aware of “how to ask friends and family members for help”, “know which government agency to call for help”, “know the nearest emergency shelter”, and “know the emergency exit.”

#### 2.2.2. Place Attachment

The place attachment was estimated by the degree of agreement to two statements: (1) “I have a sense of belonging to our community”, and (2) “I am very proud to tell others where I live.” The answers to each question ranged from one to five, indicating an increased degree of agreement to the statements. The mean value of the answers to the two questions was used to measure place attachment in this analysis.

#### 2.2.3. Self-Efficacy

Self-efficacy, also termed as one’s confidence about one’s ability to effectively engage in a behavior [33], can lead to the intention and actual perform of a disaster preparedness behavior [16]. Based on previous literature [33], we measured self-efficacy by the evaluation of the question “how do you evaluate your confidence in yourself or your family’s capacity of response if some emergency happens?”, and a five-point Likert scale measured the answers from 1 (not confident at all) to 5 (very confident) [29].

#### 2.2.4. Control Variables

Disaster experience, socioeconomic, and demographic variables were included in this analysis as the controlled variables. The disaster experience was measured by a question “Have you experienced the following disasters or emergencies in the last 10 years?” and 13 types of disasters such as earthquake, flood, landslide/debris flow, typhoon, low-temperature freezes/blizzards, droughts, water pollution, air pollution/smog, fires, large-scale infectious diseases (e.g., SARS), nuclear accidents, chemical accidents, and crowd trampling were included. The frequency of choice to each type of emergency was calculated as the experience of disasters. Based on previous literature [6,53,54,55,56,57,58], we controlled the socioeconomic and demographic variables which were potentially correlated to disaster preparedness, such as the participant’s age, whether there are children at home (yes = 1), gender (male = 1), ethnicity (Han = 1), community (rural/urban difference) (urban = 1), marital status (married = 1), Communist Party of China (CPC) membership (yes = 1), religion (yes = 1), education level (illiteracy = 0, primary = 1, middle school = 2, high school = 3, college or above = 4), annual household income (in thousand Chinese Yuan (CNY)), property ownership (yes = 1). Being a member of the Chinese Communist Party is always used as an indicator of political status and capability of acquiring resources in the Chinese context [6,57].

### 2.3. Data Analysis

We first reported the percentages of the participants’ preparedness activities and the descriptive statistics of all the variables. After that, we conducted the OLS (ordinary least squares) regressions by treating all the preparedness activities as one overall preparedness indicator and then used the material preparedness, behavioral preparedness, and awareness preparedness as separate preparedness indicators, respectively. The OLS models were used because we treated the dependent variables as continuous variables in this paper. We calculated the Cronbach’s alpha test to check the internal consistency for the concepts that included several variables. The mediation effect of self-efficacy between place attachment and preparedness was also tested using the Sobel Goodman test and the three-step test method [59,60]. All the analyses were conducted by the statistical package Stata 16 (StataCorp, College Station, Texas, TX, USA).

## 3. Results

### 3.1. Descriptive Analysis

The aggregation of all the 18 preparative activities was treated as the degree of overall preparedness. The sum of the seven material preparedness activities, the four awareness items, and the seven behavioral activities were treated as the degree of material preparedness, behavioral preparedness, and awareness preparedness, respectively [6,7]. 

As shown in Table 1, the behavioral aspects of disaster preparedness were comparatively low: only 23.99% had insurance coverage for potential emergencies, 15.51% had a reunion plan within family members for a potential emergency, 9.64% had participated in emergency training, 2.76% had been a volunteer, and 1.47% had drafted a family emergency plan. A total of 55.95% knew the nearest emergency shelter, 27.19% knew the emergency exit and how to evacuate safely, 38.59% knew which government agency to call for help during emergencies, and 75.10% knew how to ask friends and family members for help. For the four material preparedness activities, 82.25% of the participants had prepared a three-day supply of water, 59.59% had prepared a three-day supply of food, 64.90% had a flashlight, 9.90% had an emergency kit, 14.63% had a radio with batteries, 70.46% had necessary medicine for family members, and 14.59% had arranged special needs for women, children or elders.

Place attachment had a mean value of 3.72, with a standard deviation of 0.77, and a Cronbach’s alpha test result of 0.78. Self-efficacy had a mean value of 4.25, with a standard deviation of 0.87. Disaster experience ranged from 0 to 12, with a mean value of 2.33, and a standard deviation of 1.80 (Table 2).

As shown in Table 2, within the 2181 participants, 54.70% were male, 99.54% were the Han majority, 96.64% were registered as rural *Hukou*, 5.23% had religious beliefs, 78.40% of them were married, 91.29% had at least one child at home, 52.41% possessed their property right, and 7.67% were CPC members. For education degree, 23.44% of the respondents only attended primary school, 28.35% attended middle school or equivalent, 13.07% attended high school or equivalent, and 11.51% had college or above education experience. On average, the participants were 53.75 years old, and their average annual household income was about 57,190 Chinese Yuan (about 8760 US dollars).

### 3.2. Correlations between Place Attachment, Self-Efficacy, and Preparedness

This study differentiated the overall preparedness into three categories: material preparedness, behavioral preparedness, and awareness preparedness. As shown in Table 3, both self-efficacy and place attachment are correlated to the overall preparedness indicator, as well as the three different preparedness degrees, in terms of material preparedness, awareness preparedness, and behavioral preparedness.

One degree’s increase of place attachment is positively correlated with a 0.34, 0.13, 0.15, and 0.08 degree of increase in overall disaster preparedness, material preparedness, behavioral preparedness, and awareness preparedness. Thus, Hypothesis 1 is supported, demonstrating that residents with a stronger sense of place attachment prepare more for potential disasters.

Self-efficacy is also associated with a higher degree of overall disaster preparedness (β = 0.53, *p* < 0.01), material preparedness (β = 0.19, *p* < 0.01), behavioral preparedness (β = 0.12, *p* < 0.01) and the awareness preparedness (β = 0.22, *p* < 0.01). Thus, Hypothesis 2 is supported.

Moreover, the respondents that have disaster experience, with older age, male gender, CPC members, with higher education level, and with higher annual household income tend to have significantly higher levels of disaster preparedness, while the effects of variables such as having at least one child at home, ethnicity, and religious status are not significant. The results also suggest that being married, and living in an urban area tend to indicate a higher degree of material preparedness, but not behavior preparedness and awareness preparedness.

### 3.3. The Mediation Effect of Self-Efficacy

As shown in Table 3 and Table 4, place attachment was positively associated with disaster preparedness and self-efficacy. Meanwhile, self-efficacy was also significantly associated with disaster preparedness, which means the mediating effect of self-efficacy was confirmed between place attachment and all types of preparedness.

We employed the Sobel Goodman test to test the mediating effects of self-efficacy between place attachment and disaster preparedness. We estimated 2000 bootstrap samples in which the independent variable was place attachment, the mediator was self-efficacy, and the dependent variables were emergency preparedness. We also included control variables as covariates in the model. The results indicated that self-efficacy partially mediated the relationship between place attachment and overall disaster preparedness (indirect effect = 0.08; 95% CI: [0.05, 0.11]; direct effect = 0.34, 95% CI: [0.17, 0.51]). Specifically, (1) in the regression of the overall preparedness (dependent variable) and the place attachment (independent variable), the coefficient of place attachment was significant (β = 0.42, *p* < 0.01). (2) In the regression of self-efficacy (mediator) and the place attachment (independent variable), the coefficient of place attachment was significant (β = 0.15, *p* < 0.01). (3) In the regression of the overall preparedness (dependent variable) and self-efficacy (independent variable), the coefficient of mediator was significant (β = 0.53, *p* < 0.01).

**Figure 3:**
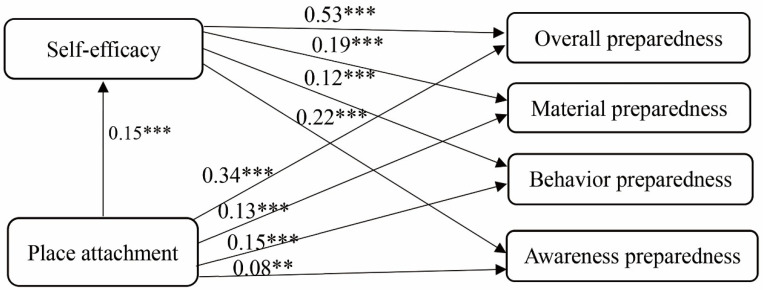
The mediating effect of self-efficacy between place attachment and preparedness. Note: *** *p* < 0.01, ** *p* < 0.05.

Similarly, we tested the mediating roles of self-efficacy between place attachment and the three types of preparedness—the material preparedness, behavioral preparedness and awareness preparedness, respectively. The results demonstrated that self-efficacy partially mediated the relationship between place attachment and material preparedness (indirect effect = 0.03; 95% CI: [0.01, 0.04]; direct effect = 0.13, 95% CI: [0.03, 0.22]), behavior preparedness (indirect effect = 0.02; 95% CI: [0.01, 0.03]; direct effect = 0.15, 95% CI: [0.07, 0.22]), awareness preparedness (indirect effect = 0.03; 95% CI: [0.02, 0.05]; direct effect = 0.08, 95% CI: [0.01, 0.15]). Three step test results of the mediating effects among material preparedness, behavioral preparedness and awareness preparedness were shown in Table 3 and Table 4. Accordingly, Hypothesis 3 was supported and the effect for each individual path was illustrated in Figure 3.

## 4. Discussion

Using representative data from Shandong province, one area that had relatively fewer occurrences of disasters but facing increasing threats of typhoon and flood recently, we analyzed the correlations between place attachment and disaster preparedness, with an effort to examine the mediating role of self-efficacy. This paper has at least the following notable contributions to the current knowledge.

Place attachment is not only positively correlated with the overall degree of disaster preparedness but is also associated with the three dimensions of disaster preparedness, namely the material preparedness, awareness preparedness, and behavioral preparedness, as we assumed in H1. Such a positive correlation is consistent with the prior investigation in India in the context of flood disasters [18], and China in the context of landslide [21], as well as Australia in the context of a wildfire [26], but contradicted the findings from Australia’s climate change adaptation [61]. One possible reason is that we did not use the multidimensional measure of place attachment, and the varied dimensions of place attachment, such as place identity, place dependence, neighborhood quality, and detachment [62], may have different or even contradicting effects on preparedness. Emotional attachment may increase people’s motivation to protect themselves and the community but make them reluctant to evacuate during emergencies. The familiarity with a community may also diminish people’s motivation to take action due to the over-confidence bias [63].

Our analysis also confirms that self-efficacy is positively correlated with disaster preparedness, as most previous studies have demonstrated. Thus, hypothesis II was supported. Moreover, we found that self-efficacy mediated the correlations between place attachment and disaster preparedness, and the path coefficients between place attachment, self-efficacy, overall preparedness, material preparedness, awareness preparedness, and behavior preparedness are statistically significant. Therefore, hypothesis III was also confirmed. Self-efficacy is one of the most critical cognitive variables that link people’s understanding of risk and the adoption of actual actions. Although some studies indicated that self-efficacy exerted more influence on planning for preparedness than actual preparedness behaviors [64], this analysis followed the same observations from Mumbai, Taiwan, and Australia [29,34,65]. Besides, we are aware that scholars have proposed several types of efficacies recently, such as the collective efficacy (how community or government can handle the potential disasters effectively) [31,32] or the responsive/outcome efficacy (how effective the actions adopted in disaster risk reduction are in reducing the impact from potential disasters) [28,29,30]. This paper contributes to our understanding that self-efficacy can directly promote disaster preparedness and play a mediation role between other variables, such as place attachment in this study and the disaster preparedness behaviors.

Additionally, we found that people with a higher level of education and being a CPC member adopted much more preparedness activities in this analysis. This finding highlighted the potential targeted vulnerable group and the household with a lower education level. It could be possible that the under-educated do not know the availability of actions they can adopt to prepare for disasters. Our previous survey about participants’ preparedness activities revealed that the majority reason for not preparing for potential disasters was that they were not aware of the existing preparedness activities. In contrast, most of the CPC members are local officials or community leaders in China, and they are usually expected to spearhead the “public desired” actions in the community. Not surprisingly, this group has a more significant potential to access the disaster risk reduction knowledge and resources, and thus, they have a much higher degree of preparedness for disaster.

The findings of this paper have practical implications for disaster risk reduction practice because it investigated the residents from an area with potential typhoons and floods, but they have not had much disaster experience previously. Considering the historical flood threats in this region and the increasing trend of typhoons and floods, this paper highlighted the importance of place attachment and self-efficacy in promoting disaster preparedness activities. Disaster risk reduction outreach programs and advocacies should and could highlight the strong sense of community and also encourage and let the public know their capacity of preparing for disasters, and thus, they can better prepare for potential hazards in the age of uncertainties.

This analysis has at least three limitations. Firstly, the inevitable limitation of the cross-sectional survey in this investigation cannot really solve the causal relations between the variables. Considering the increasing application of experiments, or experiment-embedding in surveys, studies using these new and advanced techniques could be conducted to produce more scientific conclusions in the future. Secondly, this analysis only employed data from a province with relatively fewer occurrences of disasters in China, and thus the overall generalization of this study might be needed. Thirdly, we only included limited dimensions of place attachment and efficacy measures in this analysis; studies including other dimensions of place attachment or types of efficacies such as the collective efficacy and response efficacy [28,29,30,31,32] are needed.

## 5. Conclusions

This study investigated the associations between place attachment, self-efficacy, and disaster preparedness, and we found that a stronger sense of place attachment predicts higher degrees of all the three types of preparedness, namely the material preparedness, behavioral preparedness, and awareness preparedness. Self-efficacy is also positively correlated with all types of preparedness. Moreover, self-efficacy plays a mediating role between place attachment and disaster preparedness. This study enriched the social cognitive theory in the disaster contexts by investigating the complex relationships between place attachment, self-efficacy and disaster preparedness. These findings highlight the importance of promoting self-efficacy and place attachment in disaster risk reduction advocacies and outreaches. Studies using the experimental method and covering more dimensions of the place attachment and more types of efficacy are needed in future studies.

## Figures and Tables

**Table 1:** Disaster preparedness activities.

Types	Variables	Frequency	Percentage
Material preparedness	Three-day supply of water	1793	82.25
	Three-day supply of food	1299	59.59
	Flashlight	1415	64.91
	Emergency kit	216	9.91
	Radio with batteries	319	14.63
	Necessary medicine for family members	1536	70.46
	Special needs for women, children or elders	318	14.59
Behavioral preparedness	Having a family emergency plan	32	1.47
	Having a reunion plan within family members for potential emergency	338	15.51
	Paying attention to disasters related information	908	41.69
	Purchasing accident insurance for family members	522	23.99
	Participating in emergency training	210	9.64
	Discussing with friends and family members about what to do if emergencies happened	524	24.06
	Being a volunteer or a member of community emergency response team	60	2.76
Awareness preparedness	Knowing the nearest emergency shelter	1217	55.95
	Knowing the emergency exit and how to evacuate safely	592	27.19
	Knowing which government agency to call for help during emergencies	840	38.59
	Knowing how to ask friends and family members for help	1635	75.10

**Table 2:** Descriptive statistics of independent variables.

Variables	N	Mean	SD	Min	Max
Place attachment	2172	3.72	0.77	1	5
Self-efficacy	2172	4.25	0.87	1	5
Disaster experience	2169	2.33	1.80	0	12
Age	2181	53.75	16.63	18	99
Annual household income	1863	57,190	136,786	0	4,000,000
		**Frequency**		**Percent**	
Property ownership	Yes	1143		52.41	
	No	1038		47.59	
Education	Illiteracy	511		23.44	
	Primary	515		23.62	
	Middle	618		28.35	
	High	285		13.07	
	College+	251		11.51	
Gender	Female	1193		45.30	
	Male	988		54.70	
Ethnicity	Han	2171		99.54	
	Others	10		0.46	
Community	Rural	2073		95.05	
	Urban	108		4.95	
Religion	None	2067		94.77	
	Yes	114		5.23	
Marital status	Not married	471		21.60	
	Married	1710		78.40	
CPC member	Yes	167		7.67	
	No	2011		92.33	
Child(ren) at home	Yes	1991		91.29	
	No	190		8.71	
Total		2181		100	

**Table 3:** Disaster preparedness and influencing factors (full models).

Variables	Overall Preparedness	Material Preparedness	Behavior Preparedness	Awareness Preparedness
Place attachment	0.34 ***	0.13 ***	0.15 ***	0.08 **
	(0.09)	(0.05)	(0.04)	(0.04)
Self-efficacy	0.53 ***	0.19 ***	0.12 ***	0.22 ***
	(0.08)	(0.04)	(0.03)	(0.03)
Disaster experience	0.23 ***	0.08 ***	0.10 ***	0.05 ***
	(0.04)	(0.02)	(0.02)	(0.02)
Age	−0.02 ***	0.00	−0.01 ***	−0.01 ***
	(0.01)	(0.00)	(0.00)	(0.00)
Child(ren) at home	0.13	0.12	−0.06	0.08
	(0.31)	(0.17)	(0.13)	(0.13)
Gender	0.41 ***	0.14 *	0.12 *	0.14 **
	(0.15)	(0.08)	(0.06)	(0.06)
Ethnicity	0.01	0.68	−0.23	−0.44
	(1.00)	(0.54)	(0.43)	(0.42)
Community (rural/urban)	0.31	0.33 **	0.10	−0.12
	(0.31)	(0.17)	(0.13)	(0.13)
Religion	−0.10	−0.19	0.05	0.03
	(0.30)	(0.16)	(0.13)	(0.13)
Marital status	0.49 **	0.32 ***	0.05	0.11
	(0.19)	(0.10)	(0.08)	(0.08)
Education	0.52 ***	0.13 ***	0.24 ***	0.17 ***
	(0.07)	(0.04)	(0.03)	(0.03)
CPC membership	1.23 ***	0.36 **	0.37 ***	0.51 ***
	(0.26)	(0.14)	(0.11)	(0.11)
Annual household income	0.00 **	0.00	0.00 **	0.00 ***
	(0.00)	(0.00)	(0.00)	(0.00)
Property ownership	−0.26 *	−0.13 *	−0.10	−0.03
	(0.14)	(0.08)	(0.06)	(0.06)
N	1831	1842	1840	1833
R^2^	0.19	0.07	0.16	0.16

Note: standard errors in parentheses; *** *p* < 0.01, ** *p* < 0.05, * *p* < 0.1.

**Table 4:** Test of mediating role of self-efficacy between place attachment and preparedness.

Variables	Overall	Overall	Material	Material	Behavior	Behavior	Awareness	Awareness	Self-Efficacy
Placeatt achment	0.42 ***		0.16 ***		0.17 ***		0.11 ***		0.15 ***
	(0.09)		(0.05)		(0.04)		(0.04)		(0.03)
Self-efficacy		0.58 ***		0.21 ***		0.14 ***		0.23 ***	
		(0.08)		(0.04)		(0.03)		(0.03)	
N	1835	1834	1848	1846	1844	1844	1839	1836	1842
R^2^	0.17	0.18	0.06	0.06	0.16	0.16	0.14	0.16	0.05

Note: Due to the page limitation, the results of the controlled variables were not reported here but are included in Table S1; standard errors in parentheses; *** *p* < 0.01.

## Data Availability

According to the data access policies, the data used to support the findings of this study are available from the Institute of Governance, Shandong University. Reasonable request for SGSS data is available through email: iog@sdu.edu.cn.

## Supplementary Materials

The following are available online at https://www.mdpi.com/article/10.3390/ijerph18115565/s1, Table S1: Test of mediating role of self-efficacy between place attachment and preparedness with control variables shown.
