# Supplementary material for: Place Attachment and Household Disaster Preparedness: Examining the Mediation Role of Self-Efficacy
Source: Int J Environ Res Public Health. 2021 May 23;18(11):5565. doi: 10.3390/ijerph18115565 (PMC8197108; doi:10.3390/ijerph18115565)
Supplement: Supplementary file 1 [file ijerph-18-05565-s001.zip › ijerph-1190620-supplementary.pdf]

Table 1. Test of mediating role of self-efficacy between place attachment and preparedness with control variables shown.

VARIABLES	Overall	Overall	Material	Material	Behavior	Behavior	Awareness	Awareness	Self−efficacy
Place attachment	0.42***		0.16***		0.17***		0.11***		0.15***
	(0.09)		(0.05)		(0.04)		(0.04)		(0.03)
Self−efficacy		0.58***		0.21***		0.14***		0.23***	
		(0.08)		(0.04)		(0.03)		(0.03)	
Disaster experience	0.25***	0.22***	0.09***	0.07***	0.10***	0.10***	0.06***	0.05***	0.05***
	(0.04)	(0.04)	(0.02)	(0.02)	(0.02)	(0.02)	(0.02)	(0.02)	(0.01)
Age	−0.02***	−0.02***	0.00	0.00	−0.01***	−0.01***	−0.01***	−0.01***	−0.01***
	(0.01)	(0.01)	(0.00)	(0.00)	(0.00)	(0.00)	(0.00)	(0.00)	(0.00)
Child(ren) at home	0.21	0.19	0.14	0.14	−0.05	−0.04	0.11	0.09	0.17*
	(0.31)	(0.31)	(0.17)	(0.17)	(0.13)	(0.13)	(0.13)	(0.13)	(0.09)
Gender	0.39***	0.43***	0.13	0.15*	0.11*	0.12**	0.13**	0.14**	−0.02
	(0.15)	(0.15)	(0.08)	(0.08)	(0.06)	(0.06)	(0.06)	(0.06)	(0.04)
Ethnicity	0.28	0.18	0.77	0.74	−0.17	−0.16	−0.33	−0.40	0.52*
	(1.01)	(1.00)	(0.54)	(0.54)	(0.43)	(0.43)	(0.42)	(0.42)	(0.30)
Community (rural/urban)	0.41	0.30	0.36**	0.32*	0.12	0.09	−0.08	−0.13	0.18*
	(0.31)	(0.31)	(0.17)	(0.17)	(0.13)	(0.13)	(0.13)	(0.13)	(0.09)
Religion	−0.14	−0.10	−0.20	−0.18	0.04	0.05	0.01	0.03	−0.06
	(0.30)	(0.30)	(0.16)	(0.16)	(0.13)	(0.13)	(0.13)	(0.13)	(0.09)
Marital status	0.56***	0.47**	0.35***	0.32***	0.08	0.05	0.14*	0.10	0.11*
	(0.19)	(0.19)	(0.10)	(0.10)	(0.08)	(0.08)	(0.08)	(0.08)	(0.06)
Education	0.55***	0.50***	0.14***	0.12***	0.24***	0.23***	0.18***	0.16***	0.05**
	(0.07)	(0.07)	(0.04)	(0.04)	(0.03)	(0.03)	(0.03)	(0.03)	(0.02)
CPC membership	1.30***	1.26***	0.37***	0.37***	0.38***	0.38***	0.53***	0.51***	0.10
	(0.27)	(0.26)	(0.14)	(0.14)	(0.11)	(0.11)	(0.11)	(0.11)	(0.08)
Annual household income	0.00**	0.00**	0.00	0.00	0.00**	0.00**	0.00***	0.00***	−0.00
	(0.00)	(0.00)	(0.00)	(0.00)	(0.00)	(0.00)	(0.00)	(0.00)	(0.00)
Property ownership	−0.25*	−0.24	−0.12	−0.12	−0.10	−0.09	−0.02	−0.02	0.04
	(0.15)	(0.15)	(0.08)	(0.08)	(0.06)	(0.06)	(0.06)	(0.06)	(0.04)
Observations	1,835	1,834	1,848	1,846	1,844	1,844	1,839	1,836	1,842
R−squared	0.17	0.18	0.06	0.06	0.16	0.16	0.14	0.16	0.05

Note: standard errors in parentheses; *** $p < 0.01$, ** $p < 0.05$, * $p < 0.1$;
